# Novel Roles for MLH3 Deficiency and TLE6-Like Amplification in DNA Mismatch Repair-Deficient Gastrointestinal Tumorigenesis and Progression

**DOI:** 10.1371/journal.pgen.1000092

**Published:** 2008-06-13

**Authors:** Peng-Chieh Chen, Mari Kuraguchi, John Velasquez, Yuxun Wang, Kan Yang, Robert Edwards, Dan Gillen, Winfried Edelmann, Raju Kucherlapati, Steven M. Lipkin

**Affiliations:** 1 Department of Biological Chemistry, University of California Irvine, Irvine, California, United States of America; 2 Department of Medicine, University of California Irvine, Irvine, California, United States of America; 3 Department of Genetics, Harvard Medical School, Harvard Partners Center for Genetics and Genomics, Boston, Massachusetts, United States of America; 4 Department of Medicine, Brigham and Women's Hospital, Boston, Massachusetts, United States of America; 5 Department of Molecular Genetics, Albert Einstein College of Medicine, New York, New York, United States of America; 6 Strang Cancer Research Laboratory, Department of Medicine, Weill Medical College of Cornell University, New York, New York, United States of America; 7 Department of Pathology, University of California Irvine, Irvine, California, United States of America; 8 Department of Statistics, University of California Irvine, Irvine, California, United States of America; Stanford University School of Medicine, United States of America

## Abstract

DNA mismatch repair suppresses gastrointestinal tumorgenesis. Four mammalian *E. coli* MutL homologues heterodimerize to form three distinct complexes: *MLH1/PMS2*, *MLH1/MLH3*, and *MLH1/PMS1*. To understand the mechanistic contributions of *MLH3* and *PMS2* in gastrointestinal tumor suppression, we generated *Mlh3^−/−^*;*Apc^1638N^* and *Mlh3^−/−^*;*Pms2^−/−^*;*Apc^1638N^* (*MPA*) mice. *Mlh3* nullizygosity significantly increased *Apc* frameshift mutations and tumor multiplicity. Combined *Mlh3*;*Pms2* nullizygosity further increased *Apc* base-substitution mutations. The spectrum of *MPA* tumor mutations was distinct from that observed in *Mlh1^−/−^*;*Apc^1638N^* mice, implicating the first potential role for *MLH1/PMS1* in tumor suppression. Because *Mlh3*;*Pms2* deficiency also increased gastrointestinal tumor progression, we used array-CGH to identify a recurrent tumor amplicon. This amplicon contained a previously uncharacterized *Transducin enhancer of Split* (*Tle*) family gene, *Tle6-like*. Expression of *Tle6-like*, or the similar human *TLE6D* splice isoform in colon cancer cells increased cell proliferation, colony-formation, cell migration, and xenograft tumorgenicity. *Tle6-like*;*TLE6D* directly interact with the gastrointestinal tumor suppressor *RUNX3* and antagonize *RUNX3* target transactivation. *TLE6D* is recurrently overexpressed in human colorectal cancers and *TLE6D* expression correlates with *RUNX3* expression. Collectively, these findings provide important insights into the molecular mechanisms of individual MutL homologue tumor suppression and demonstrate an association between *TLE* mediated antagonism of *RUNX3* and accelerated human colorectal cancer progression.

## Author Summary

Approximately one million people every year are diagnosed with colorectal cancer worldwide, and about five hundred thousand of these people subsequently perish from the disease. Colorectal cancer is thought to develop through a series of early and later stages (called cancer initiation and progression, respectively). Deaths from colorectal cancer are particularly tragic because the disease can usually be cured if discovered before full-blown progression. However, our knowledge of how these tumors progress remains very limited. DNA mismatch repair is known to be an important process in preventing ∼15% of colorectal cancer initiation. In this study we describe how two of these genes (Mlh3 and Pms2) that have partial functional redundancy and therefore individually are rarely mutated are also important in preventing colorectal cancer progression. Additionally, we describe a new gene (Tle6-like) that, when overactive, makes these cancers progress more rapidly. The overall goal of this study is to understand colorectal cancer progression better so that we can come up with new ways to block it at the later stage.

## Introduction

Colorectal cancer (CRC) is one of the common malignancies in industrialized countries. Lynch syndrome, a highly penetrant disorder that confers predisposition to cancer of the colorectum, endometrium and other extra-colonic sites [1], is caused by germline mutations in DNA Mismatch Repair genes (MMR). Including sporadic forms, defective MMR underlies ∼12–15% of CRC [2]. MMR plays critical roles in the maintenance of genomic stability in both prokaryotes and eukaryotes [3]. The study of model organisms has yielded great insights into the mechanisms through which MMR prevents cancer [1],[3],[4],[5],[6],[7],[8]. Briefly, there are nine mammalian MMR genes (*MLH1*, *MLH3*, *PMS1-2*, *MSH2-6*). The mammalian E *coli* MutS homologues (MSH) directly contact DNA, scanning along the genomic DNA for mismatches analogous to a “sliding clamp” until they encounter a base-pair containing a mismatch [9],[10]. MSH2-MSH6 primarily recognizes single-base substitutions and 1 base-pair insertion-deletion loop (IDL) mutations, while MSH2-MSH3 recognizes 1–4 base-pair insertion-deletion mutations [1],[3].The IDL repair deficiency is commonly referred to as Microsatellite Instability (MSI). The MSH proteins interact with multiple proteins including the mammalian E *coli* MutL homologues (MLH) and yeast post-meiotic segregation (PMS) homologue proteins (which have significant amino acid identify and structural similarity to the MLH proteins), as well as *RPA*, *EXO1*, *RFC*, *HMGB1*, *POLDC* and other proteins [1],[8],[11],[12]. MLH1-PMS2 is the primary MutL complex that interacts with both MSH2/6 and MSH3 complexes. MLH1–MLH3 is less well characterized, but is believed to participate in IDL repair [13],[14], DNA damage response [13], and possibly single-base point mutation repair (SBR)[15]. MLH1-PMS1 exists in mammalian cells but currently has no clearly defined roles in processes related to cancer prevention [16],[17].

To study the precise mechanisms through which MMR suppresses carcinogenesis *in vivo*, we and others [16],[18],[19],[20],[21],[22],[23],[24] previously developed several mouse models carrying mutations in different MMR genes. *Mlh1^−/−^* and *Msh2^−/−^* mice develop early onset GI epithelial cancers, lymphomas and other types of cancer. *Pms2^−/−^* mice develop lymphomas, but not GI epithelial cancers. *Mlh3^−/−^* mice develop GI and extra-GI tumors, have decreased survival when compared with *Wt* mice, but with later onset than *Mlh1^−/−^* [13]. *Mlh3^−/−^*;*Pms2^−/−^* mice have increased cancer incidence, resistance to apoptosis and MSI [13]. However, the precise mechanisms in which *Mlh3* and *Pms2* participate to suppress GI epithelial tumorigenesis and progression remain poorly characterized.

Germ-line mutations in tumor suppressor gene *APC* lead to familial adenomatous polyposis (FAP) [25],[26]. Mutations in *APC* are found in the majority of sporadic CRC and many Lynch syndrome tumors [27],[28]. APC complexes with AXIN and CK1/2 and destabilizes β-Catenin by enhancing proteasomal destruction. Mutated *APC* proteins are unable to down-regulate β-Catenin, and the stabilized β-Catenin translocates into the nucleus where it acts as a transcriptional coactivator of the DNA binding protein TCF-4 [29],[30]. More than 95% of *APC* germ-line mutations are truncating or nonsense mutations and most of the pathogenic mutations are located within the first 1500 codons. *Apc* mutations cooperate with MMR deficiency in both tumorigenesis and tumor progression. *Apc^1638N^* mice are a well characterized model that develops GI cancer [31]. *Mlh1^−/−^*;*Apc^1638N^* mice showed significantly increased GI tumor multiplicity and accelerated progression to adenocarcinoma compared to either mutation separately. Analyses of GI tumors from *Mlh1^−/−^*;*Apc^1638N^* and *Msh3^−/−^*;*Msh6^−/−^*;*Apc^1638N^* mice revealed that both single-base substitutions and MSI induced frameshift mutations in repetitive sequences were responsible for most mutations found in the remaining wild-type (Wt) *Apc* allele [32],[33]. In contrast, tumor-associated *Apc* mutations found in the Wt *Apc* allele in *Msh6^−/−^*;*Apc^1638N^* tumors were predominantly single-base point mutations.

To understand more precisely the mechanistic roles that *Mlh3* and *Pms2* play in GI tumor suppression, we generated *Mlh3^−/−^*;*Apc^1638N^* (*MA*) and *Mlh3^−/−^*;*Pms2^−/−^*;*Apc^1638N^* (*MPA*) mice. We show that *in vivo Mlh3* mutations significantly increase frameshift mutation rates in *Apc*, and increase GI tumorigenesis. Unlike typical MSI-induced mutations, *Mlh3* deficiency also results in frameshift mutations in non-repetitive sequences, a unique mutational signature among MMR deficient mice found only in *Mlh3* deficient mice. Consistent with the role of *Pms2* in SBR, combined *Mlh3* and *Pms2* mutations proportionally increase point mutations and show a sequence preference for a *CpG* mutation hotspot also previously seen in *Mlh1^−/−^* mice. Because *MPA* mutant mice also have significantly increased rates of GI adenocarcinomas vs. *Apc^1638N^* or *MA* mice, we investigated mechanisms of tumor progression. Using array-CGH, we identified a recurrent 5-Mb amplification on chromosome 12 in GI tumors from *MPA* mice. We defined the amplicon critical interval and demonstrated that it contains a previously uncharacterized member of the *Transducin enhancer of Split* (*TLE*)/*Groucho* family of transcriptional co-regulators, *Tle6-like*, that contributes to tumor progression. *Tle6-like* overexpression in colon cancer cell lines increases cell proliferation, colony-formation ability, cell migration and xenograft tumorigenicity. Human *TLE6D*, an alternatively spliced isoform of *TLE6*, with a domain structure similar to *Tle6-like*, has functional activity similar to *Tle6-like*. Both *Tle6-like* and *TLE6D* interact with GI tumor suppressor, *RUNX3* [34], and antagonize *RUNX3* gene target tranactivation. *TLE6D* is overexpressed in multiple human microsatellite stable (MSS) and microsatellite unstable (MSI-H) CRCs, and *TLE6D* expression levels correlate with *RUNX3* expression levels. Collectively, these findings provide important insights into the molecular mechanisms through which MMR-deficiency contributes to GI tumorigenesis and implicate a novel association between *TLE6* isoforms and antagonism of *RUNX* target gene expression in CRC tumor progression.

## Results

### *Mlh3*, *Pms2* and *Apc* Mutations Cooperate to Increase Tumor Incidence, Accelerate Progression and Decrease Overall Survival

**Figure 1:**
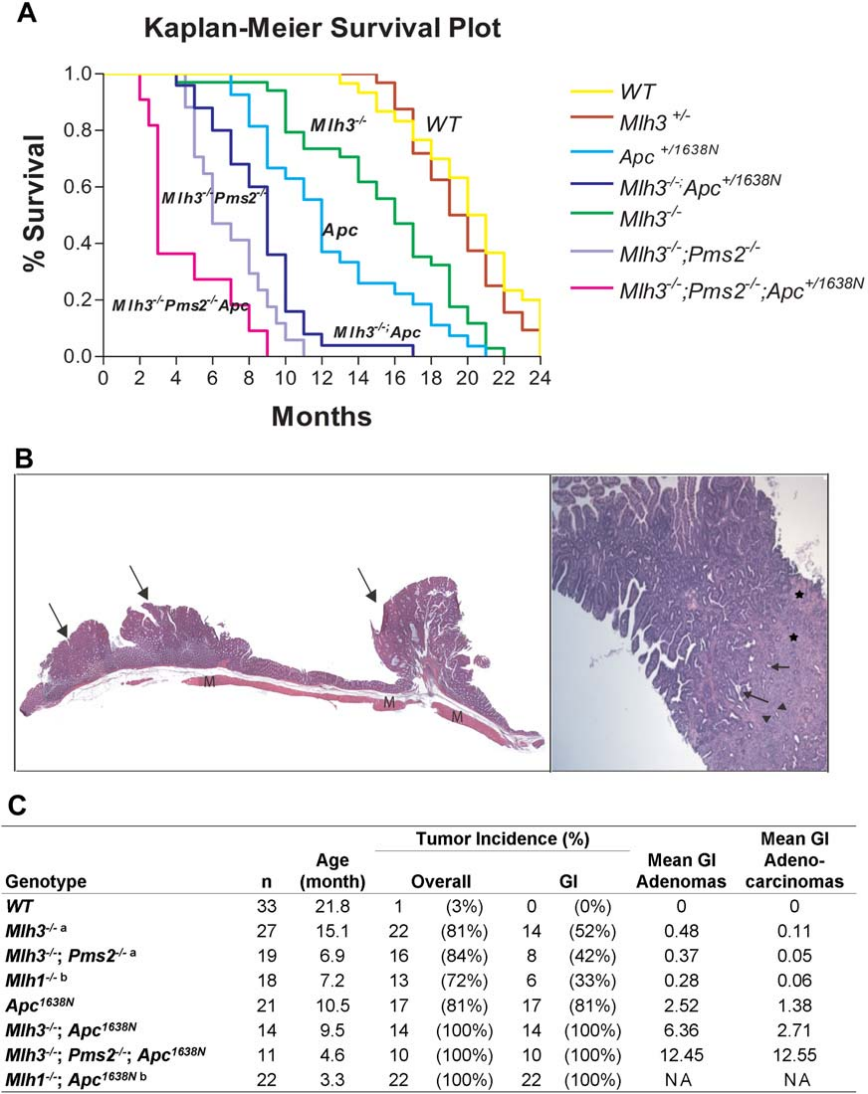
Decreased survival and increased tumor multiplicity and progression in *MPA* mice. (A) Kaplan-Meier survival analysis of mice deficient in Apc and DNA Mismatch Repair genes. (B, left) Three small intestinal adenomatous polyps from MPA mouse duodena are seen in cross-section (arrows, left panel). (B, right panel) Example of a jejeunal adenocarcinoma from an MPA mouse, showing atypical glands (arrows) invading deeply into a desmoplastic stroma (asterisks) containing a mixed chronic inflammatory reaction (arrowheads). (C) Tumor incidence and multiplicity. ^a^ Data from Chen et al[13]. ^b^ Data from Edelmann *et al* [35], for comparison. NA, data not available.

By 9.5 months of age, *MA* mice develop >50% more tumors than *Apc^1638N^* mice (P<0.001; Mann-Whitney) (Figure 1A and C). However, the relative ratios of GI adenomas to carcinomas in *Apc^1638N^* mice (65% and 35% respectively) were very similar to that seen in *MA* mice (70% and 30% respectively) and overall survival is not significantly affected (9.5 vs. 10.5 months). No significant effect was seen on extra-GI cancer incidence or progression. These data suggest the primary role of *Mlh3* is in suppression of GI tumor initiation and not tumor progression.

To study the effects of combined *Mlh3* and *Pms2* mutations *in vivo*, we generated *MPA* mice. *MPA* mice had significantly shorter survival vs. *Apc^1638N^* or *MA* mice (P<0.01, Mann-Whitney test; Figure 1A, C) and developed significantly more adenocarcinomas than *MA* or *Apc^1638N^* mice (Figure 1B, C) (P = 0.022 *MPA* vs. *MA* and p = 0.0003 *MPA* vs. *Apc^1638N^*). These are consistent with a role for *Mlh3*;*Pms2* combined loss both to increase GI tumor initiation and accelerates progression. However, mean overall survival of MPA mice is longer than that previously seen in *Mlh1^−/−^*;*Apc^1638N^* mice [35].

### Spectrum of *Apc* Mutations in *MA* and *MPA* Mice Tumors

**Figure 2:**
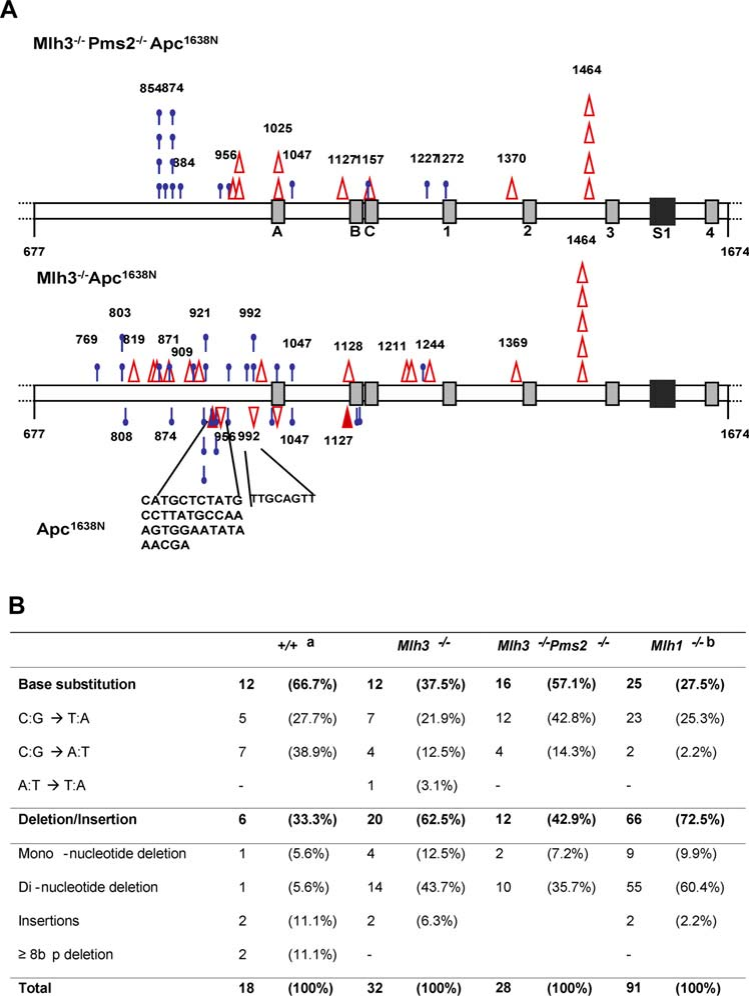
Distribution of *Apc* mutations. (A) Diagram of *Apc* between codons 677 and 1674 showing positions and characteristics of truncation mutations detected in *MPA (top)*, *MA* (middle) and *Apc^1638N^* (bottom) gastrointestinal tumors. (Open triangle symbol, deletion; solid triangle, insertion; blue bar, substitution). Each symbol represents an independent mutation. Note the common hotspots at position 1464 in both strains. The three 15-aa (A–C), four 20-aa (1–4) β-catenin binding repeats and one SAMP repeat in this segment of Apc are indicated. The two nuclear export signals are at the ends of the third and fourth 20-aa repeats. (B) Spectrum of *Apc* truncation mutations in MMR deficient Apc^1638N^ tumors. ^a^ Combined with previous data from Wong et al, 2002. ^b^ Data from Kuraguchi et al, 2000, for comparison.

*In vitro* studies have alternatively suggested that *Mlh3* participates in either IDL repair [13] or SBR [15]. To understand the role of *Mlh3* in these processes, we used the wild type *Apc* allele as a tumor-associated *in vivo* reporter gene to analyze the mutation spectrum from *MA* GI tumors. A total of 49 tumors from *MA* mice and 28 tumors from *Apc^1638N^* littermates were analyzed for *Apc* truncation mutations by IVTT analysis. Truncated Apc products were detected in 27 of 49 (55%) *MA* tumors while only 9 of 28 (32%) were found in *Apc^1638N^* tumors. The current observed incidence of *Apc* somatic mutations of *Apc^1638N^* tumors is in agreement with the previous results (7 of 22, 32%) [36], hence for better understanding of mutational differences between the two strains, this and the previous data for *Apc^1638N^* tumors were combined and used for further comparisons. This 23% increase in somatic *Apc* mutations in *MA* mice was significant (P<0.0048; Fisher exact test) and was attributable to increased small insertion/deletion frameshift mutations (62.5%) vs. *Apc^1638N^* (33.3%) mice (P<0.001; Fisher exact test; Figure 2B and Tables 1 and 2). *MA* mice had one recurrent insertion/deletion mutation “hotspot” also observed in *Mlh1*;*Apc^1638N^* mice (amino acid 1464) (Figure 2A). Furthermore, examination of the sequences surrounding each *Apc* mutation site in *MA* tumors showed that, unlike in other mismatch repair deficient tumors such as *Mlh1^−/−^*;*Apc^1638N^* or *Msh6^−/−^*;*Msh3^−/−^*;*Apc^1638N^* [32],[33], about 40% of frameshift mutations occurred at non-repetitive sequences within the *Apc* coding region. These data are consistent with a primary *in vivo* role for *Mlh3* in DNA repair of small insertion/deletion mutations in GI epithelial cells.

**Table 1:** *Apc* truncation mutations in intestinal tumors from MutL homolog deficient Apc^1638N^ mice.

	+/+ Apc^1638N^ a	Mlh3^−/−^ Apc^1638N^	Mlh3^−/−^ Pms2^−/−^ Apc^1638N^	Mlh1^−/−^ Apc^1638N^ b
Tumor DNA samples analyzed	50 (100%)	49 (100%)	49 (100%)	44 (100%)
Tumors samples with Apc truncations detected by IVTT	16 (32%)	27 (55%)	25 (51%)	37 (84%)
Tumor samples showing >1 mutant allele	2 (4%)	5 (10%)	4 (8.1%)	29 (66%)
Total *Apc* mutations characterized by sequencing	18 (100%)	32 (100%)	28 (93%)	91 (100%)

a Combined with previous data from Wong *et al* 2002 [36].

b Data from Kuraguchi *et al* 2000 [32] for comparison.

**Table 2:** Sequences of *Apc* mutations in MutL homolog deficient Apc^1638N^ tumors.

Codon	Mutation	Conseq- uence	Wild-type Sequence ^b^	Apc^1638N^	Mlh3^−/−^ Apc^1638N^	Mlh3 Pms2^−/−^ Apc^1638N^
769	G→T	Glu→Stop	TTA TCA **G**AA ACC TTC	-	1	-
778	ΔT	frameshift	AAC CTA AG**T** CCC AAG	-	-	-
803	C→T	Arg→Stop	GCC AAT **C**GA CAT GAT	-	2	-
808	ΔG	frameshift	GAT AGT AG**G** TCA GAC	1	-	-
819	ΔTG	frameshift	ATG AC**T G**TT CTT TCA	-	1	-
847	ΔAC	frameshift	GAG AAA G**AC** AGA AGT	-	1	-
848	ΔAG	frameshift	AAA GAC **AG**A AGT TTG	-	1	-
853	ΔAG	frameshift	GAG AGA G**AG** CGA GGT	-	1	-
854	C→T	Arg→Stop	GAG AGA GAG **C**GA GGT	-	1	4
866	G→T	Glu→Stop	ACA ACA **G**AA AAT GCA	-	-	1
871	ΔTC	frameshift	GGA ACC **TC**A TCA AAA	-	1	-
872	C→A	Ser→Stop	ACC TCA T**C**A AAA CGA	-	-	-
874	C→T	Arg→Stop	TCA AAA **C**GA GGT CTG	1^a^	-	4
884	C→T	Gln→Stop	GCA GCC **C**AG ATA GCC	-	-	1
902	ΔAG	frameshift	GAC GAC **AG**A AGT TCT	-	1	-
909	G→T	Glu→Stop	ACC ACC **G**AG TTC CAT	-	1	-
913	ΔTG	frameshift	CAT TGT G**TG** GCA GAC	-	1	-
921	C→T	Arg→Stop	GCG GCA **C**GA AGA AGC	3^a^	2	-
933	C→A	Tyr→Stop	AAC ACA TA**C** AAC TTC	1	-	-
934	+TACA	frameshift	AAC ACA **TAC A**AC TTC	1^a^	-	-
939	G→T	Glu→Stop	AAG TCG **G**AA AAT TCA	2	-	1
944	Δ38bp	frameshift	TCAAATAGGA**CATGCTCTATGCCTTATGCCAAAGTGGAATATAAACGA** TCT TCA AAT	1	-	-
956	C→T	Arg→Stop	TAT AAA **C**GA TCT TCA	1	1	1
959	ΔA	frameshift	TCT TCA A**A**T GAC AGT	-	-	1
974	ΔAA	frameshift	GGT AA**AA**GA GGC CAA			1
974	ΔGA	frameshift	GGT AAA A**GA** GGC CAA			1
984	T→A	Tyr→Stop	GAA TCC TA**T** TCT GAA	-	1	-
992	+T	frameshift	AAA TTT **T**GC AGT TAT	-	2	-
992	Δ8bp+A	frameshift	AAA TT**T TGC AGT T**AT	1^a^	-	-
1004	ΔC	frameshift	GAC CTA GCC **C**AT AAG	-	1	-
1018	G→T	Glu→Stop	GAT GGA **G**AA CTG GAT	1	-	-
1025	C→A	Tyr→Stop	ATA AAT TA**C** AGT CTT	-	1	-
1025	ΔAC	frameshift	ATA AAT T**AC** AGT CTT	1	-	2
1047	G→A	Trp→Stop	GAA AGG TG**G** GCA AGA	1^a^	1	1
1127	+T	frameshift	CAG TCT C**T**G TGT CAG	1	-	-
1127	ΔCT	frameshift	CAG TCT **CT**G TGT CAG			1
1128	ΔGT	frameshift	TCT CTG T**GT** CAG GAA	-	1	-
1141	C→A	Tyr→Stop	ACC AAC TA**C** AGT GAA	1	-	-
1143	G→T	Glu→Stop	TAC AGT **G**AA CGT TAT	1	-	-
1154	G→T	Glu→Stop	GAA GAA **G**AA GAA GAG			1
1157	ΔGA	frameshift	GAA GAG A**GA** CCG ACA			1
1211	ΔTC	frameshift	CAT CTC TC**T C**CA AGC	-	1	-
1219	ΔG	frameshift	ACA GCT **G**TA CCT CCA	-	1	-
1227	C→T	Gln→Stop	AAA AGG **C**AG AAT CAG	-	-	1
1272	C→A	Cys→Stop	TCA AGG TG**C** AGT TCA	-	-	1
1234	C→A	Ser→Stop	CCA AGT T**C**A GCA CAA	-	1	-
1244	ΔG	frameshift	CAA AAA G**G**C ACT ACT	-	1	-
1370	ΔA	frameshift	ACA CCC AAA **A**GT CCC	-	1	1
1464	ΔAG	frameshift	GAG AAG AGA GAG **AG**T	-	5	4
			Total	18	32	28

a Previous data from Wong et al 2002.

We also studied the tumor-associated *Apc* mutations in GI tumors from *MPA* mice. The overall incidence of *Apc* truncation mutations in *MPA* tumors were similar to that observed in *MA* tumors, yet the nature of mutations characterized was distinct. Compared with *MA* mice (37.5%), combined *Mlh3*;*Pms2* deficiency caused a significant increase in the proportion of single-base point mutations (57.2%, P<0.01; Figure 2 and Table 2). Within the types of single-base point mutations, *MPA* tumors showed higher frequency of C∶G→T∶A transition mutations (12 of 16, 75%) compared to *MA* tumors (7 of 12, 58.3%). However, this high frequency was not as prominent as that of *Mlh1*;*Apc^1638N^* tumors which showed the majority (23 of 25, 92%) of base substitutions to be transition mutations[32]. The C∶G→T∶A transition mutations found in tumors, irrespective of genotypes, occurred at either *CpG* dinucleotides or CpNpG sites, typical targets for DNA methylation. Among these, *Apc* codon R854 seems to be a preferential target for base substitution mutation, which was not only demonstrated to be a mutational hotspot in *Mlh1*;*Apc^1638N^* mice [32] but also in *MPA* mice.

### Identification of Genomic Copy Amplification in *MPA* Tumors Associated with Tumor Progression

**Figure 3:**
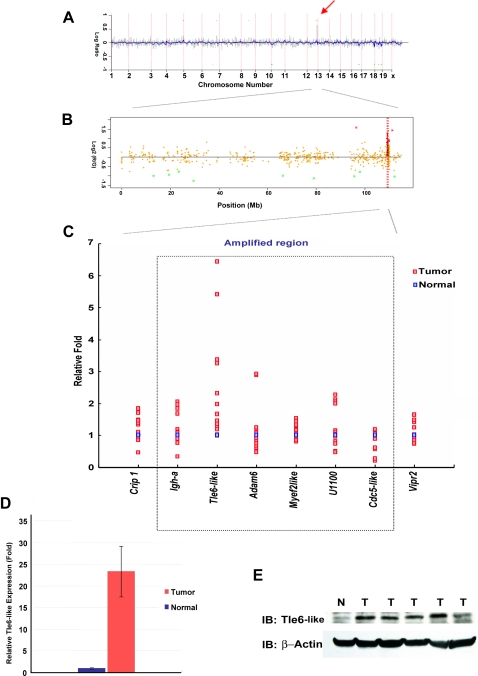
Array-CGH analysis of *Mlh3*;*Pms2*;*Apc* deficient GI tumors. (A) Display of CGH signal from a representative tumor genome wide. Red arrow indicates the gain of signal on chromosome 12. (B) Higher resolution view of mouse chromosome 12 signal. Red dot line indicates the amplification. (C) Quantitative PCR of genomic DNA level from *MPA* tumors. Dotted box indicates the amplified region detected by array-CGH. (D) Quantitative PCR of *Tle6-like* level in cDNA from *MPA* tumors. (E) Immunoblot of Tle6-like in *MPA* tumors. N, normal tissues; T, tumors.

**Figure 4:**
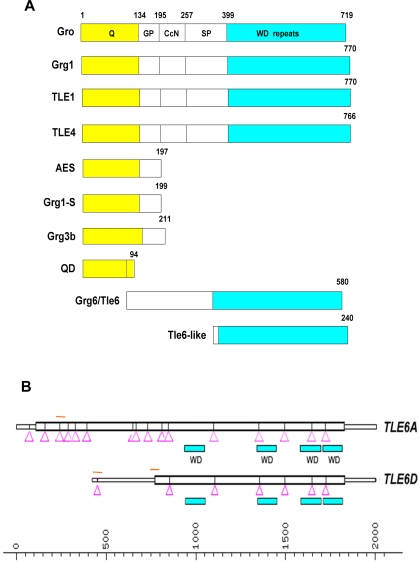
TLE Family. Schematic diagram of TLE protein family members. (A) Numbers indicate amino acids. Q, glutamine rich domain; GP, Glycine/praline rich domain; CcN, domain containing putative phosphorylation sites and putative nuclear localization signal; SP, serine/praline rich domain; WD repeats, domain containing series of tandem repeats of tryptophan and aspartic acid residues. (B) Diagram of *TLE6* RNA and alternative splice form *TLE6D* is indicated. *TLE6A* is the full length mRNA transcript. Orange bars indicate position of primer sets for qPCR. Arrows indicate exon-exon junctions.

*Apc* mutation is thought to be an early event in CRC carcinogenesis. The significantly increased number of adenocarcinomas vs. adenomas seen in *MPA* vs. *MA* or *Apc^1638N^* mice suggested that *MPA* tumors have accelerated tumor progression. While there is extensive evidence that increased mutation rates and decreased apoptosis contribute to MMR defective CRC, it is likely that additional mechanisms participate in tumor progression as well. Because chromosomal and segmental aneuploidy has been described in a subset of MMR deficient adenocarcinomas [37],[38],[39], we performed array comparative genomic hybridization (aCGH) analyses of GI tumor vs. E18.5 C57BL/6 embryonic control DNA from *Apc*, *MA*, and *MPA* mice to identify specific genetic changes that accelerate *MPA* GI tumor progression. Comparison of aCGH profiles revealed a recurrent 5-Mb base pairs amplification on chromosome 12F2 (66.7%∼83.3%; see Table 3 for detail; Figure 3A and B) in *MPA* GI tumors not seen in *Apc^1638N^* or *MA* tumors (Figure S1). To define the critical interval for this amplification on chromosome 12F2 we bred a new cohort of *MPA* mice and quantified copy number variation in the tumor using real-time quantitative PCR (qPCR) (Figure 3C and Table 4). Using qPCR with primer sets for the six genes within the amplified region and two flanking genes, we identified one gene that showed recurrent increased level of genomic DNA in tumor tissues (Figure 3C), *Transducin-like enhancer protein 6-like*, *(Tle6-like*). *TLE* family members act as transcriptional corepressors [40],[41] without any intrinsic DNA-binding activity. They are recruited to specific gene regulatory sequences in a context-dependent manner by forming complexes with different DNA-binding transcription factors. Two evolutionarily conserved domains define the *TLE* gene family: an N-terminal glutamine-rich (Q) domain that mediates *TLE* family member heterodimerization, and a C-terminal domain of WD motif repeats that mediates direct interactions with sequence specific DNA binding transcription factors (Figure 4)[40],[41]. Previously *TLE* family members have been described containing only the Q domain, such as *Grg1-S* [42], or only the WD repeat motif, such as *Grg6/Tle6* [43]. *Tle6-like* similarly contains only the C-terminal WD repeat domain and had highest amino acid identity (84.4%) with *TLE6* (Figure 4A and Figure S2).

**Table 3:** Amplifications of Chromosome 12.

Chromosome	Change	Start Position	Start Band	End Position	End Band	Frequency
12	Gain	109,044,957	12 q F1	109,106,314	12 q F1	8 /12 (66.7%)
12	Gain	109,106,314	12 q F1	109,348,509	12 q F1	10/12 (83.3%)
12	Gain	109,348,509	12 q F1	109,556,449	12 q F1	9/12 (75%)

**Table 4:** Primers Used in Real-Time PCR.

Primer	Sequences
Crip-F	GGCTGCCACATTGAAAGAAT
Crip-F	TCAGCTGCAGAAGCACAGAT
Cdc5L-F	TGGCAATATATGCTGTCTTGTAGG
Cdc5L-F	TGCCTCTTCCTCAAAGTCCT
Myef2l-F	CATGGTCAGGCCTATCACAA
Myef2l-F	GACTTCCCTTGGTCATGGTG
Tle6_F	ACACTATCTTAGGCCTCAAGTTCTCTC
Tle6_R	AGTCATGCCATAGCATCTGACAGT
Adam6-F	CACCTGCATCATGTTCAAAAA
Adam6-R	GACATGGCATCAGATCAGGA
Igh-a_F	AGCAGTCTGAGGTCTGAGGACACGGCC
Igh-a_R2	TGCTCTTCAGGAGGTTTTAGTT
U110086690_F	ATGGAATGGAGTTGGGTCTTT
U110086690_R	TTTGTCATCGCAGACCCTGT
Vipr2_F	GTGAGCAGCATCCATCCAG
Vipr2_R	CCTCTCTGATTCTCCGTTTGG
Alkbh-F	GTAATGCCTCCCAGAAGTGC
Alkbh-R	CTGCTGAGCTGGTGAAATTG

### RNA and Protein Expression Levels of *Tle6-like* Are Increased in *MPA* Tumors

To understand the impact of gene amplification on *Tle6-like* expression, we isolated total RNA from tumor and normal tissues from *MPA* mice and used qPCR to quantify relative *Tle6-like* mRNA expression. As a result of copy number amplification, *Tle6-like* mRNA levels were significantly increased in tumors compared with adjacent normal GI tissue (Figure 3D). To understand whether *Tle6-like* protein levels are subsequently increased, we generated anti-Tle6-like specific antisera. Western blot analysis with this antisera demonstrated that *Tle6-like* protein levels are significantly increased in GI tumors compared to surrounding normal GI epithelial tissue from *MPA* mice (Figure 3E). Overall, these data suggest increased genomic DNA copy number of *Tle6-like* causes increased mRNA and protein expression of *Tle6-like* in *MPA* tumors.

### Expression Level of *TLE6* Alternative Spliced Isoform D (*TLE6D*) Is Increased in Human Colorectal Tumors

**Figure 5:**
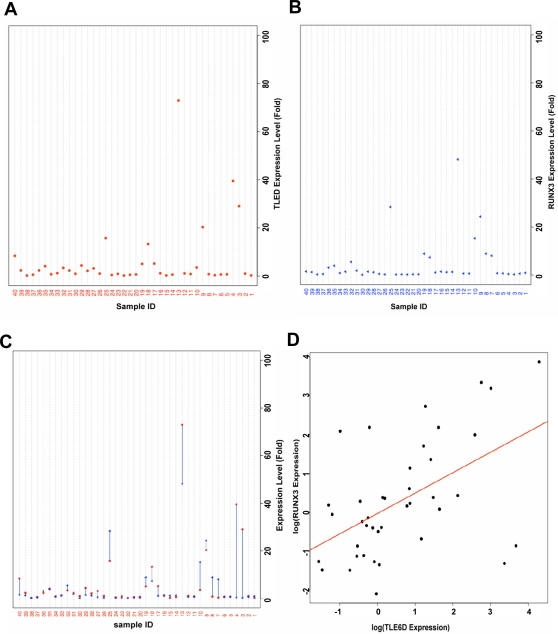
TLE6D and *RUNX3* mRNA expression levels in human colorectal cancers. (A,B) Dotplots of *TLE6D* and *RUNX3* expression levels for each of the 40 samples. Expression is shown as fold elevation vs. accompanying normal adjacent mucosa. (C) Superimposed plot of *TLE6D* and *RUNX3* expression levels by sample (Pearson correlation 0.724; Pvalue<0.001). (D) A scatterplot of log-transformed *RUNX3* by *TLE6D* expression levels along with the least squares estimate of the regression of *RUNX3* on *TLE6D*. Expression levels were log-transformed in the regression analysis due to heavy skewness. Based upon the regression, it was estimated that the geometric mean of *RUNX3* expression increased 0.525 with a 2-fold increase in *TLE6D* expression (95% CI: 0.165, 0.563; p Value<0.001).

Gene diversity can be generated by several mechanisms, including gene duplication and paralogue evolutionary divergence, and the generation of alternative mRNA splice isoforms that modify coding sequence. The mouse *Tle6-like*-containing amplicon is syntenic to human chromosome 14q33, but amplification of this chromosomal region is not associated with CRC. Upon further analysis, we discovered that 14q33 contains no human ortholog of mouse *Tle6-like*, or any other *TLE* family member. However, when we analyzed *TLE6* mRNAs bioinformatically, we identified a previously identified alternative spliced isoform of *TLE6 (TLE6D)* (Genbank Accession #BX375733) that contains only the C-terminal WD repeat domain of *TLE6*, and therefore has the same domain structure as mouse *Tle6-like* (Figure 4B) To understand expression of *TLE6A* (full-length isoform) and *TLE6D* in human CRC, we generated three sets of RT-PCR primers: one for the *TLE6D* N-terminus, one crossing the splice junction that is specific for *TLE6D* and one that detects *TLE6A* but not *TLE6D* (Figure 4B). We then calculated expression of these transcripts in 40 human CRC samples and normal tissue. Compared to adjacent normal tissue, the *TLE6D*-specific and *TLE6* C-terminus qPCR showed significantly increased expression in a subset of human CRCs (Figure 5A), but not for the *TLE6* N-terminal or *TLE6A* qPCR (data not shown). These data suggest that the *TLE6D* isoform specifically is overexpressed in a subset of human CRCs.

### *Tle6-like* and *TLE6D* Enhance Cell Proliferation, Colony Formation, and Cell Migration

**Figure 6:**
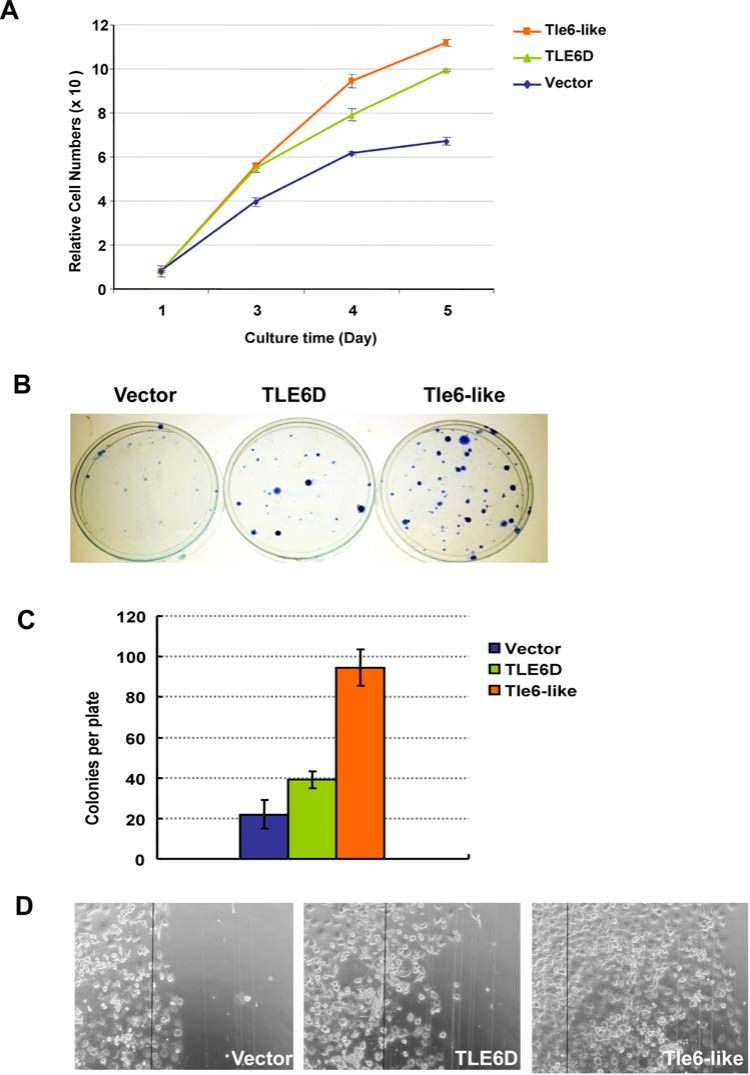
Tle6-like and *TLE6D* Enhance Cell Proliferation, Colony Formation, and Cell Migration. (A) HCT116 Cell proliferation MTT assay. (B) Representative picture of plates of colony formation assay on MEFs transfected with vector, *TLE6D*, and *Tle6-like*. (C) Plot of number of colonies from colony formation assay. (D) *In Vitro* Cell Mobility Assay. “Wound” was generated by razor blade, clearing the adherent cells on the right side of the slides. Black lines indicate the edge of the “wound”. Representative pictures from HCT116 cells transfected with vector, *TLE6D*, and *Tle6-like* are shown.

Because GI tumors from *MPA* mice showed increased number of adenocarcinoma than *Apc^1638N^* or *MA* mice, we evaluated whether increased levels of *Tle6-like* can contribute to mechanisms that underlie tumor progression. We generated stable cell 293 cell lines that express *Tle6-like* or *TLE6D*. For both *Tle6-like* and *TLE6D* overexpressing cell lines, cell proliferation rates were significantly increased compared with vector-transfected control cells (Figure 6A). Similar results were also seen in HCT116 and 3T3 cells (data not shown). We next tested the effect of *Tle6-like*/*TLE6* expression on the ability to form colonies *in vitro*. Mouse embryonic fibroblasts transfected with *Tle6-like* or *TLE6D* significantly increased colony formation (four-fold and two-fold, respectively) compared with empty vector-transfected control cells (Figure 6B and C). We also tested the mobility of the cells transfected with *Tle6-like/TLE6D* by *in vitro* migration assay. Cell lines stably expressing *Tle6-like* or *TLE6D* were able to migrate a significantly longer distance when compared with control cell lines expressing only the vector (Figure 6D). In contrast, no effect of *Tle6-like* or *TLE6D* ectopic expression was seen on induction or resistance of apoptosis induced by serum-depletion in culture medium (data not shown). In summary, these results are consistent with a proliferation and migration advantage for tumor cells expressing *Tle6sh* or *TLE6D*.

### *Tle6-like* and *TLE6D* Expression Increases Xenograft Tumor Proliferation *in vivo*

**Figure 7:**
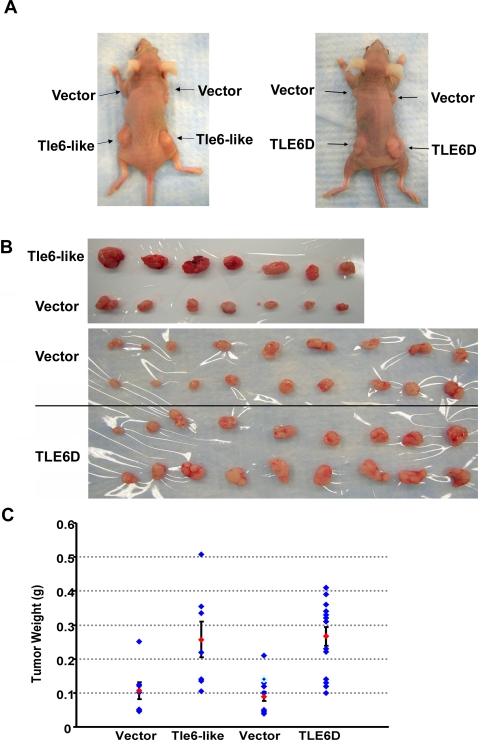
Tle6-like and *TLE6D* Enhance Gastrointestinal Tumor Progression. (A) Left panel, Xenograft of HCT116 cell lines expression *Tle6-like* or vector alone. Right Panel, Xenograft of HCT116 cell lines expression *TLE6D* or vector alone. (B) Representative xenograft tumors of HCT116 cells expressing *pCS2+MT* -*Tle6-like*, *pCS2+MT* vector, *pcDNA6/HisA-TLE6D* and empty *pcDNA6/HisA* vector. (C) Tumor weight in HCT116 xenograft tumors expressing *Tle6-like* or empty *pCS2+MT* vector and *TLE6D* and empty *pcDNA6/HisA* vector (Error bars indicate the standard deviation of the mean; p Value<0.005).

Because *Tle6-like* or *TLE6D* ectopic expression increased cell proliferation and migration *in vitro*, we evaluated their impact *in vivo*. We injected HCT116 cells stably expressing *Tle6-like*, *TLE6D* or vector s.c. into nude mice and quantified tumor growth. As expected, HCT116 cells transfected with vector formed xenograft tumors. In parallel, HCT116 cells expressing *Tle6-like* and *TLE6D* formed significantly larger tumors (Figure 7). These results suggest that *Tle6-like* and *TLE6D* expression increases CRC cell proliferation and growth, *in vivo*.

### *Tle6-like* and *TLE6D* Interact with the *RUNX3* GI Tumor Suppressor and Antagonize *RUNX3* Mediated Transactivation

**Figure 8:**
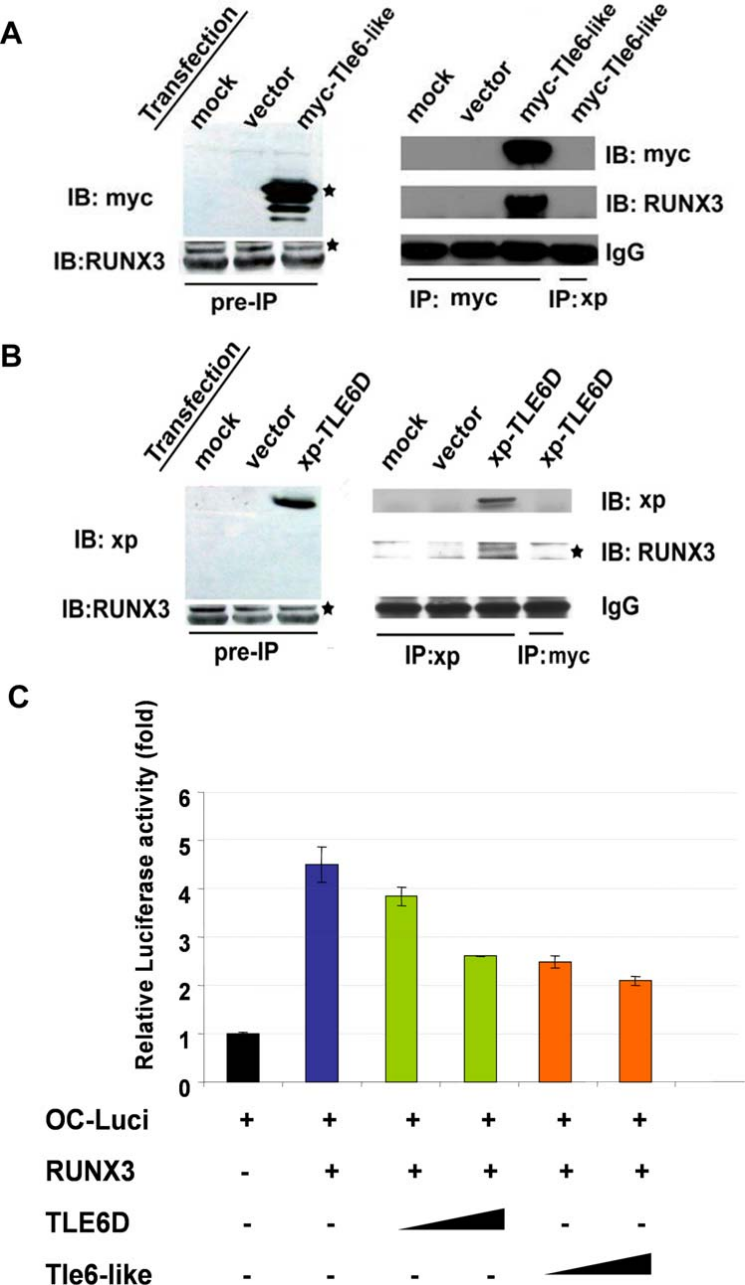
Tle6-like and *TLE6D* Antagonize *RUNX3* Gene Target Transcription. (A and B) Tle6-like/TLE6D interacts with endogenous RUNX3 in 293 cells. Left panel represents the immunoblot of protein extracts before immunoprecipitation. Right panel represents the immunoblot after the immunoprecipitation. (C) Luciferase reporter assay. Cells were transient tranfected with indicated plasmids and relative luciferase activities were determined the next day. (Error bars indicate the standard deviation of the mean).

*RUNX* genes encode transcription factors that activate or repress transcription of key regulators of growth, survival and differentiation pathways [44],[45]. This gene family is defined by the Runt domain, which mediates both protein-DNA and protein-protein interactions with transcriptional co-regulators. *TLE* proteins interact with, and regulate the function of, *RUNX* proteins through direct interactions between the *TLE* WD domain and the Runt domain and the interactions antagonize *RUNX*-mediated transactivation [44],[45],[46],[47],[48]. *RUNX3* has been shown to play important roles in GI epithelial cell development and tumorgenesis. Loss of *Runx3* predisposes knockout mice to gastric hyperplasia, indicating a tumor suppressor-like role for this gene [34],[49],[50],[51],[52]. In human gastric cancers, hypermethylation of *RUNX3*, hemizygous deletion and truncating point mutations have been observed [34],[52],[53],[54],[55],[56],[57],[58]. To test whether Tle6-like/TLE6D interact with RUNX3, we first evaluated sub-cellular localization using immunofluorescence staining in 293 cells co-transfected with *Tle6-like* or *TLE6D* and native *RUNX3* (Figure S3). Using anti-Myc, anti-Xpress and anti-RUNX3 antibodies, we observed that highest levels of Tle6-like and TLE6 and are in the nucleus overlapping with nuclear RUNX3 staining. Furthermore, in 293 cells, transiently transfected with *Tle6-like* or *TLE6D*, endogenous RUNX3 co-immunoprecipitated with anti-Myc or anti-Xpress antibodies (Figure 8A and B), suggesting an interaction between Tle6-like/TLE6D and RUNX3. Similar co-localization and co-immunoprecipitation results were seen in HCT116 and 3T3 cells (data not shown). Finally, to evaluate the functional consequences of Tle6-like/TLE6D interaction on RUNX3 transcriptional regulation we used a well characterized *RUNX3* transactivation on promoter target, osteocalcin (OC), fused to a luciferase reporter gene [47]. As expected, transfected *RUNX3* activated luciferase expression in 293, Hela or HCT116 cells (Figure 8C, lane 1 and 2). Co-transfection of *Tle6-like* or *TLE6D* decreased *RUNX3* transcriptional reporter activity in a dose-dependent manner (Figure 8C), whereas *Tle6-like*/*TLE6D* transfection had no effect on promoters lacking *RUNX3* binding sites, such as the TOPFLASH/FOPFLASH system (data not shown). Taken together, these results are consistent with a model whereby *Tle6-like/TLE6D* expression antagonizes *RUNX3* GI tumor suppressor mediated target gene transactivation through an interaction between the Tle6-like/TLE6D and RUNX3, providing a selective growth advantage for cell proliferation and migration.

### *TLE6D* is Highly Expressed in Human CRCs with High *RUNX3* Expression Levels

In gastric cancer, *RUNX3* activity is most commonly reduced through a mechanism involving *RUNX3* promoter hypermethylation and subsequently decreased mRNA expression. However, its expression levels in CRC have not been well characterized. We therefore used qPCR to evaluate *RUNX3* expression in 40 human CRC and matched normal GI epithelial samples, normalized to *GAPDH* expression. In many CRCs, *RUNX3* expression is low, consistent with a role in GI tumor suppression. However, in a subset of CRCs *RUNX3* expression is paradoxically increased (Figure 5B). To test whether elevated *TLE6D* expression is associated with *RUNX3* activation, we used qPCR to analyze *TLE6D* expression levels in the same matched sets of CRCs and normal mucosa. We observed a clear correlation of *RUNX3* and *TLE6D* expression levels (R = 0.723; Figure 5C). However, at the same time no clear correlation was seen for *RUNX3* and *TLE6D* expression levels with regard to MSI-H/MSS status or for expression levels of the full length *TLE6* and *RUNX* (data not shown). Overall, in combination with the functional antagonism of *RUNX3* activity by *TLE6D* observed in colon cancer cells, the correlation of *RUNX3* and *TLE6D* expression in human CRCs suggests that *TLE6D* may interact with the *RUNX3* GI epithelial tumor suppressor and inactivate *RUNX3* in a subset of CRCs independent of MSI status. However, further experiments will be required to analyze the association between RUNX3 and TLE6D expression levels and functional interactions in more detail.

## Discussion

Because *APC* is a common mutation target in MMR-deficient CRC, we created novel mouse models combining different mutations in these genes to analyze their roles in MMR-deficient GI carcinogenesis and progression. The observation that *MA* mice have increased tumor multiplicity but no accelerated tumor progression or decreased survival vs. *Apc^1638N^* mice suggests a primary role for the Mlh1–Mlh3 heterodimer in suppression of GI tumor initiation. While previous *in vitro* studies have alternatively suggested that Mlh1–Mlh3 participates in IDL repair [13] and SBR[15],[59], our study provides the first *in vivo* evidence that *Mlh3* deficiency significantly increases IDL mutation frequency. This type of mutation occurred both at repetitive and non-repetitive *Apc* sequences, implicating its role in repair of both types of IDL (Figure 2). Previous studies of *Pms2^−/−^*;*Apc^Min^* mice have shown a primary role for Mlh1-Pms2 in GI tumorgenesis suppression but not tumor progression[60]. We therefore combined these mutations to create *MPA* mice. Like *Mlh1^−/−^*;*Apc^1638N^* mice, *MPA* mice have significantly increased GI tumor multiplicity, accelerated tumor progression and decreased overall survival[61] . *MPA* tumors harbor proportionally more C∶G→T∶A (at either CpG or CpNpG sites) transition mutations than *MA* tumors, showing recurrence in certain arginine codons, one of which was at *Apc* codon 854, a SBR hotspot that was also previously seen in *Mlh1^−/−^*;*Apc^1638N^* mice.

In addition to Mlh1-Pms2 and Mlh1–Mlh3, several lines of evidence from our study suggest a potential role for Mlh1-Pms1 in suppression of GI tumorigenesis. First, *MPA* mice have later mean GI tumor onset compared to previous studies of *Mlh1^−/−^*;*Apc^1638N^* mice[32]. Second, the multiplicity of GI tumors is decreased vs *Mlh1^−/−^*;*Apc^1638N^* mice. Third, two *Apc* insertion/deletion mutation hotspots seen in *Mlh1^−/−^*;*Apc^1638N^* mice have not been detected in *MPA* tumors. These data are consistent with previous studies of yeast *Mlh2p* (orthologue of mammalian *PMS1*) that demonstrate a minor role for this protein in IDL repair [62].

Because the combination of *Mlh3*, *Pms2* and *Apc* mutations accelerates tumor progression, we searched *MPA* GI tumor specific genetic changes associated with progression using high-resolution aCGH. *MPA* tumors contained a recurrent 5-Mb amplicon with a critical interval containing a novel, poorly characterized member of the *TLE* family of transcriptional co-repressors, *Tle6-like*. Unexpectedly, this *MPA* recurrent amplification hotspot is not detected by aCGH in GI tumors from *Mlh1^−/−^*;*Apc^1638N^* mice (data not shown). The reason for this difference is unclear, but again suggests that Mlh1-Pms1 may play a role in causing chromosomal instability.

*TLE* genes are the mammalian homologues of *Drosophlia groucho* that play critical roles in a wide range of developmental and cellular pathways [40]. TLE proteins are transcriptional corepressors for specific families of DNA-binding transcription factors, including RUNX proteins[48]. In addition, *Tle1/Grg1* has been shown to act as a lung-specific oncogene in a transgenic mouse model [63]. Mouse *Tle6/Grg6* has been shown to synergize with the E2A-HLF oncoprotein in antagonism of *Runx1* transactivation in murine pro-B cells, causing acute leukemogenesis [64]. *Tle6/Grg6* also participates in developmental mechanisms of neurogenesis [43]. Here, we provide data that a previously uncharacterized TLE family member containing only the WD repeat domain, *Tle6-like*, has amplified gene copy number, mRNA and protein levels in GI epithelial tumors from MMR deficient/*Apc* mutant mice, and is associated with accelerated tumor progression. Consistent with this observation, in functional studies *Tle6-like/TLE6D* enhances cell proliferation, colony-formation, migration and xenograft tumorgenicity. While *TLE* family members have previously been shown to repress *Wnt/*β*-catenin* signaling [42],[65],[66],[67], we were unable to demonstrate any *Tle6-like/TLE6D* protein-protein interactions with β*-catenin* or effect of *Tle6-like/TLE6D* overexpression on β*-catenin* reporter gene activity using TOPFlash in transient transfection in colon cancer cell lines (data not shown), suggesting that *Tle6-like/TLE6D* might not be involved in canonical Wnt pathway.

*RUNX* family genes regulate lineage and stage specific gene transcription by direct binding to DNA promoters and enhancer elements [44],[45]. Loss of *Runx3* in the mouse results in the development of gastric mucosal hyperplasia, decreased apoptosis and attenuated *TGF-*β anti-proliferative signaling. Consistent with previous observations of interactions between RUNX3 and TLE family members mediated through the Runt and WD repeat domains, respectively [46],[48], we detected an interaction between RUNX3 and Tle6-like/TLE6D by co-immunoprecipitation. Furthermore, we demonstrated that Tle6-like/TLE6D antagonized RUNX3 regulated transcriptional targets. However, while these experiments show an association between RUNX3∶TLE6D interactions and tumor progression, they do not demonstrate mechanistically the functional importance of this interaction in accelerating tumor progression.

Alternative mRNA splicing allows multiple gene products to be produced from a single coding sequence, and through this mechanism a higher diversity of mammalian genes is generated [68]. Several distinct *TLE/Grg* gene alternative splice forms, such as *Grg-1s*, *QD of TLE4*, and *Grg3b* [42],[69],[70], have been reported. While the human genome does not encode a *TLE6-LIKE* ortholog, a structurally equivalent protein, *TLE6D*, is generated through alternative splicing. The observation that GI adenocarcinomas from both humans and mice use two very distinct mechanisms to amplify *Tle6-like/TLE6D* activity suggests a strong growth advantage and selective pressure for this TLE isoform in tumor progression. Similarly, the correlation between *TLE6D* and *RUNX3* expression in human CRC suggests a model whereby *RUNX3* inactivation by *TLE6D* could be an important factor driving this growth advantage in both MSI-H and MSS CRC. Future studies will be required to understand the mechanistic implications of the interaction between these two proteins in CRC progression in more precise detail.

## Materials and Methods

### Mouse Lines and Survival Analyses, Tumor Analysis, and *Apc* Mutation Analysis

Wild-type (*Wt*), *Pms2^+/−^* and *Mlh3^+/−^* mice were maintained on the 129 Sv/Ev genetic background and intercrossed to generate *Mlh3^+/−^*;*Pms2^+/−^* mice as described before [13]. *Apc^1638N^* mice were backcrossed four times to 129 Sv/Ev and subsequently intercrossed with *Mlh3^+/−^*; *Pms2^+/−^* to generate *Mlh3^−/−^*;*Apc^1638N^* and *Mlh3^−/−^*;*Pms2^−/−^*;*Apc^1638N^* mice. Kaplan-Meier survival curves were generated and statistical significance between genotypes was determined using the Log Rank test as previously performed [13]. All lines of mice were necropsied when they became morbid or moribund. Sacrificed mice were surveyed for tumors and suspicious masses were histology analyzed as previously performed. Statistical analyses of tumor onset and incidence among the different mouse lines were performed using the Mann-Whitney test as previously described [23],[32],[33],[35],[71],[72],[73],[74],[75],[76]. Tumors from stomach, small intestine, and colon were cut into two parts. One part of the tumor was processed for histopathological analysis and the other part was used for DNA/RNA extractions. Genomic DNA samples were extracted using Puregene DNA Isolation kit (Gentra Systems, Minneapolis, MN) and subjected to mutational analysis of *Apc* gene between codons 677–1674 as previously described [33].

### Array Comparative Genomic Hybridization Analysis

Genomic DNAs were isolated from tumor tissue and tail tissue from each mouse using PUREGENE DNA Isolation kit (Gentra Systems, Minneapolis, MN). DNAs were digested with *Dpn*II and subsequently purified using the QIAquick PCR Purification kit (Qiagen). The quality of the DNA samples was evaluated using the Agilent 2100 BioAnalyzer. The purified fragmented DNA samples were random-prime labeled with either Cy5 or Cy3 and hybridized as previously described [77]

Briefly, for each labeling reaction, 2 µg of purified digested DNA were used. Each sample was dye-swap labeled for hybridization to mouse 70-mer oligonucleotide microarrays (Agilent Technologies, Palo Alto, CA) containing 20,281 clones. After hybridization, the arrays were scanned using an Agilent Microarray DNA scanner (Agilent Technologies) and the spot intensity was extracted from slide images using Agilent Feature Extraction Software 7.0. The data were further analyzed using the procedures of Automatic Data Analysis Pipeline (ADAP). Only spots with fluorescence intensities statistically different from the surrounding background (*P*<0.001) were considered reliable, taking up >85% of total spots on the chip. For further analysis the fluorescence intensity values of reliable spots were transformed to log2. To minimize the effect of the variations, the log2 intensity ratios of remaining spots were subjected to normalization by Lowess fitting. Gene copy number changes for each sample was calculated by taking the median of the normalized log2 intensity ratios of dye-swapped chip experiments for the corresponding sample. The gene copy numbers were ordered along chromosomes by the map positions of corresponding genes. To eliminate systematic noise, gene copy number changes (log2Ratios) along the chromosomes were smoothed by taking a moving median of symmetric 5-nearest neighbors, followed by Lowess fitting (f = 0.2). The mean and standard deviation (SD) of smoothed log2Ratios for all genes in all the samples were calculated. The copy number profiles of at least 5 consecutive genes that deviated significantly above mean+3SD were interpreted as regional gains, below mean-3SD as regional losses. The threshold for whole chromosomal gain/loss was mean±2SD. The ideograms of chromosomal aberrations were drawn using mapping information of cytogenetic bands to the mouse genome (NCBI Mapview Build 32).

### cDNA Preparation and Real-Time PCR

For RNA extractions, Trizol reagent (Invitrogen) was used to isolate total RNA. RNA were further digested with RNAse-free DNAseI (Promega) and cleaned with RNeasy Mini kit (Qiagen). High Capacity cDNA Archive kit from Applied Biosystems was used to make cDNA from the RNA samples. Real-time quantitative PCR was performed with either SYBRGreen PCR master mix or Taqman PCR master mix (Applied Biosystems) following the manufacture's protocol on ABI 7900 machine. Primers used for SYBR Green assays are listed in Table 1. Each gene was normalized to the internal control gene *Gapdh* and then compared to a known single copy gene (*Alkbh*), which is located on non-amplified region on chromosome 12 D3 in the *MPA* tumors.

### Generation of Tle6-Like Antibody

The whole *Tle6-like* gene (encoded 240 amino acids) was cloned in to pET28b vector and Tle-6like protein was induced and purified from *E. coli*. Rabbit anti-serum was raised against Tle6-like protein. The anti-serum was further purified using affinity column, in which Tle6like protein was covalently bound to CNBr-activated Sepharose 4B (Sigma). The purified antibody was used in immunoblotting at 1∶100 dilutions.

### Cell Culture

HCT116, 293, Hela or 3T3 cells were maintained in DMEM with 10%FBS and transfected using Lipofectamine 2000 (Invitrogen). The human isoform *TLE6D* cDNA clone was purchased from Invitrogen (Full-length Human Clones CS0DC017YC05; Accession number BX375733). *Tle6-like* was cloned from cDNA samples from *MPA* mice. We subcloned *Tle6-like* and *TLE6D* into either Xpress-epitope-tagged pcDNA6/HisA vector (Invitrogen) or Myc-tagged pCS2+MT vector. Cells were transfected with following plasmids: *pcDNA6/HisA*, *pcDNA6/HisA-Tle6-like*, *pcDNA6/HisA-TLE6D*, *pCS2+MT*, *pCS2+MT-Tle6-like*, *pCS2+MT-TLE6D*. Stable cell lines from each transfectant were generated with the selection medium containing 10 µg/ml blasticidin (Calbiochem) for 10 days. The pooled populations of cells that survived were used in the experiments for MTT assay and cell mobility assay. The transient-transfected cells were used for colony formation assay, immunoprecipitation, and reporter assay.

### MTT Cell Proliferation Assay and Colony Formation Assay

For the cell proliferation assay, 4000 cells were plated in 96-well plates and MTT assay were used to determine the cell numbers in a time-course experiment. Briefly, cells were washed with PBS and treated with 5 µg/ml MTT ([3-(4,5-dimethylthiazol-2-yl)- diphenyltetrazolium bromide]Sigma, St. Louis, MO) for 5 hours. After removal of MTT, DMSO was added to dissolve the dark purple formazam crystals in the viable cells and absorbance of 600 nm were determined by a multiwell scanning spectrophotometer. The cell numbers were calculated with a control standard curve. For colony-formation assay, MEF cells were seeded in 6 well plates and transient-transfected with 1 µg of the respective plasmids in the next day. After 24 h, cells were trypsinzed, transferred to 10-cm plates and allowed to grow with the selection medium containing 10 µg/ml blasticidin for 2 weeks. Survived cells were fixed in 30% ethanol and stained with 0.25% methylene blue. Colonies containing more than 50 cells were counted. Both assays were repeated three times in three independently-derived cell lines.

### *In Vitro* Cell Mobility Assay

The monolayer “wounding assay” was used to demonstrate the *in vitro* cell migration. Human colon cancer HCT116 cells stably expressing corresponding plasmids were plated on glass microscopy slides and cultured to confluence. A “wound” was generated by scratching the slide with a razor blade, clearing a portion of adherent cells on the slide. Photo documentation was taken at day 4 and the migration of cells from the cut edge of the monolayer into the clear portion of the slides was assessed. Two independently-derived stable cell lines for each plasmid were used in this assay.

### Antibodies, Immunoprecipitation, and Immunoblotting

Transient-transfected 293 cells in 10-cm plate were lysed with 1 ml of NP-40 lysis buffer and prepared as described before [13]. Five hundred µl of lysates were pre-cleared with 50 µl ProteinA/G agarose beads (Santa Cruz) for 1 h. After spinning down the ProteinA/G beads, the collected supernatants were incubated with 5 µg anti-Xpress or anti-myc monoclonal antibody (Invitrogen) and 50 µl ProteinA/G beads overnight at 4°C. The next day, the beads were washed with NP-40 buffer 5 times and incubate with 4× protein loading dye (Invitrogen) 10 min at 95°C to elute the binding proteins. These samples were resolved by SDS-PAGE and the immunoblotting was used as previously described to detect the corresponding proteins. The antibodies used in immunoblotting are: mouse monoclonal anti-Xpress and anti-myc (1∶2000, Invitrogen), rabbit anti-RUNX3 (1∶1000, Abcam) and goat anti-β-actin (1∶1000, Santa Cruz Biotechnologe).

### Luciferase Reporter Assay

293, Hela or 3T3 cells were transient-transfected accordingly with the *Flag-RUNX3* (a kind gift from Dr. Yoshiaki Ito) and rat Osteocalcin promoter fused to luciferase reporter construct (OC-Luci, a kind gift from Dr. Gary Stein), and plasmids as described above. Luciferase activities were determined using Dual-Luciferase reporter assay systems kit (Promega) on the luminemeter.

### Tumor Growth in Nude Mice

Female 6-week-old nude mice (Charles River Laboratories, Wilmington, MA) were divided into four experimental groups, five for each. One million HCT116 cells stably transfected with vectors (*pCS2+MT* or *pCDNA6/HisA*), *pCS2+MT-Tle6sh*, or *pCDNA6/HisA-TLE6D* were injected subcutaneously in the flanks of each mice. Mice were monitored daily for palpable tumors. Because of rapid growth, tumors were dissected out 3 weeks after injection and were analyzed.

## Supporting Information

Figures S1 Array Comparative Genome Hybridization (aCGH) analysis of GI tumors. (A) Display of aCGH signal genome wide from a representative *Apc1638N* tumor. (B) Display of aCGH signal genome wide from a representative *Mlh3−/−*;*Apc1638N* tumor. (C) Display of aCGH signal genome wide from a representative *Mlh3−/−*;*Pms2−/−* tumor.
(0.07 MB PPT)

Figure S2 Protein sequences of TLE family.
(1.95 MB PNG)

Figure S3 Cellular localization of endogenous *RUNX3* and transfected Myc-epitope tagged *Tle6-like* in 293cells. Mouse monoclonal anti-myc and rabbit anti-RUNX3 were used. Secondary FITC-conjugated anti-mouse and Cy5-conjuaged anti-rabbit antibodies were used respectively. DAPI (4′,6-diamidino-2-phenylindole) staining indicates the nuclear location.
(0.19 MB PPT)

## References

[1] Kunkel TA, Erie DA (2005). DNA mismatch repair.. Annu Rev Biochem.

[2] Ribic CM, Sargent DJ, Moore MJ, Thibodeau SN, French AJ (2003). Tumor microsatellite-instability status as a predictor of benefit from fluorouracil-based adjuvant chemotherapy for colon cancer.. N Engl J Med.

[3] Kolodner RD, Marsischky GT (1999). Eukaryotic DNA mismatch repair.. Curr Opin Genet Dev.

[4] Edelmann L, Edelmann W (2004). Loss of DNA mismatch repair function and cancer predisposition in the mouse: animal models for human hereditary nonpolyposis colorectal cancer.. Am J Med Genet.

[5] Marcon E, Moens PB (2005). The evolution of meiosis: recruitment and modification of somatic DNA-repair proteins.. Bioessays.

[6] Neuberger MS, Di Noia JM, Beale RC, Williams GT, Yang Z (2005). Somatic hypermutation at A.T pairs: polymerase error versus dUTP incorporation.. Nat Rev Immunol.

[7] Stojic L, Brun R, Jiricny J (2004). Mismatch repair and DNA damage signalling.. DNA Repair (Amst).

[8] Muller A, Fishel R (2002). Mismatch repair and the hereditary non-polyposis colorectal cancer syndrome (HNPCC).. Cancer Invest.

[9] Fishel R (2001). The selection for mismatch repair defects in hereditary nonpolyposis colorectal cancer: revising the mutator hypothesis.. Cancer Res.

[10] Acharya S, Foster PL, Brooks P, Fishel R (2003). The coordinated functions of the E. coli MutS and MutL proteins in mismatch repair.. Mol Cell.

[11] Constantin N, Dzantiev L, Kadyrov FA, Modrich P (2005). Human mismatch repair: reconstitution of a nick-directed bidirectional reaction.. J Biol Chem.

[12] Dzantiev L, Constantin N, Genschel J, Iyer RR, Burgers PM (2004). A defined human system that supports bidirectional mismatch-provoked excision.. Mol Cell.

[13] Chen PC, Dudley S, Hagen W, Dizon D, Paxton L (2005). Contributions by MutL homologues Mlh3 and Pms2 to DNA mismatch repair and tumor suppression in the mouse.. Cancer Res.

[14] Flores-Rozas H, Kolodner RD (1998). The Saccharomyces cerevisiae MLH3 gene functions in MSH3-dependent suppression of frameshift mutations.. Proc Natl Acad Sci U S A.

[15] Cannavo E, Marra G, Sabates-Bellver J, Menigatti M, Lipkin SM (2005). Expression of the MutL homologue hMLH3 in human cells and its role in DNA mismatch repair.. Cancer Res.

[16] Prolla TA, Baker SM, Harris AC, Tsao JL, Yao X (1998). Tumour susceptibility and spontaneous mutation in mice deficient in Mlh1, Pms1 and Pms2 DNA mismatch repair.. Nature Genetics.

[17] Raschle M, Marra G, Nystrom-Lahti M, Schar P, Jiricny J (1999). Identification of hMutLbeta, a heterodimer of hMLH1 and hPMS1.. J Biol Chem.

[18] Lipkin SM, Moens PB, Wang V, Lenzi M, Shanmugarajah D (2002). Meiotic arrest and aneuploidy in MLH3-deficient mice.. Nat Genet.

[19] Baker SM, Bronner CE, Zhang L, Plug AW, Robatzek M (1995). Male mice defective in the DNA mismatch repair gene PMS2 exhibit abnormal chromosome synapsis in meiosis.. Cell.

[20] de Wind N, Dekker M, Berns A, Radman M, te Riele H (1995). Inactivation of the mouse Msh2 gene results in mismatch repair deficiency, methylation tolerance, hyperrecombination, and predisposition to cancer.. Cell.

[21] Reitmair AH, Cai JC, Bjerknes M, Redston M, Cheng H (1996). MSH2 deficiency contributes to accelerated APC-mediated intestinal tumorigenesis.. Cancer Res.

[22] Reitmair AH, Redston M, Cai JC, Chuang TC, Bjerknes M (1996). Spontaneous intestinal carcinomas and skin neoplasms in Msh2-deficient mice.. Cancer Res.

[23] Edelmann W, Umar A, Yang K, Heyer J, Kucherlapati M (2000). The DNA mismatch repair genes Msh3 and Msh6 cooperate in intestinal tumor suppression.. Cancer Research.

[24] Edelmann W, Yang K, Umar A, Heyer J, Lau K (1997). Mutation in the mismatch repair gene Msh6 causes cancer susceptibility.. Cell.

[25] Groden J, Thliveris A, Samowitz W, Carlson M, Gelbert L (1991). Identification and characterization of the familial adenomatous polyposis coli gene.. Cell.

[26] Kinzler KW, Nilbert MC, Su LK, Vogelstein B, Bryan TM (1991). Identification of FAP locus genes from chromosome 5q21.. Science.

[27] Huang J, Zheng S, Jin SH, Zhang SZ (2004). Somatic mutations of APC gene in carcinomas from hereditary non-polyposis colorectal cancer patients.. World J Gastroenterol.

[28] Konishi M, Kikuchi-Yanoshita R, Tanaka K, Muraoka M, Onda A (1996). Molecular nature of colon tumors in hereditary nonpolyposis colon cancer, familial polyposis, and sporadic colon cancer.. Gastroenterology.

[29] Gregorieff A, Pinto D, Begthel H, Destree O, Kielman M (2005). Expression pattern of Wnt signaling components in the adult intestine.. Gastroenterology.

[30] Reya T, Clevers H (2005). Wnt signalling in stem cells and cancer.. Nature.

[31] Smits R, Kartheuser A, Jagmohan-Changur S, Leblanc V, Breukel C (1997). Loss of Apc and the entire chromosome 18 but absence of mutations at the Ras and Tp53 genes in intestinal tumors from Apc1638N, a mouse model for Apc-driven carcinogenesis.. Carcinogenesis.

[32] Kuraguchi M, Edelmann W, Yang K, Lipkin M, Kucherlapati R (2000). Tumor-associated Apc mutations in Mlh1−/− Apc1638N mice reveal a mutational signature of Mlh1 deficiency.. Oncogene.

[33] Kuraguchi M, Yang K, Wong E, Avdievich E, Fan K (2001). The distinct spectra of tumor-associated Apc mutations in mismatch repair-deficient Apc1638N mice define the roles of MSH3 and MSH6 in DNA repair and intestinal tumorigenesis.. Cancer Res.

[34] Li QL, Ito K, Sakakura C, Fukamachi H, Inoue K (2002). Causal relationship between the loss of RUNX3 expression and gastric cancer.. Cell.

[35] Edelmann W, Yang K, Kuraguchi M, Heyer J, Lia M (1999). Tumorigenesis in Mlh1 and Mlh1/Apc1638N mutant mice.. Cancer Res.

[36] Wong E, Yang K, Kuraguchi M, Werling U, Avdievich E (2002). Mbd4 inactivation increases Cright-arrowT transition mutations and promotes gastrointestinal tumor formation.. Proc Natl Acad Sci U S A.

[37] Campbell MR, Wang Y, Andrew SE, Liu Y (2006). Msh2 deficiency leads to chromosomal abnormalities, centrosome amplification, and telomere capping defect.. Oncogene.

[38] Sinicrope FA, Rego RL, Halling KC, Foster N, Sargent DJ (2006). Prognostic impact of microsatellite instability and DNA ploidy in human colon carcinoma patients.. Gastroenterology.

[39] Trautmann K, Terdiman JP, French AJ, Roydasgupta R, Sein N (2006). Chromosomal instability in microsatellite-unstable and stable colon cancer.. Clin Cancer Res.

[40] Stifani S, Blaumueller CM, Redhead NJ, Hill RE, Artavanis-Tsakonas S (1992). Human homologs of a Drosophila Enhancer of split gene product define a novel family of nuclear proteins.. Nat Genet.

[41] Jennings BH, Pickles LM, Wainwright SM, Roe SM, Pearl LH (2006). Molecular recognition of transcriptional repressor motifs by the WD domain of the Groucho/TLE corepressor.. Mol Cell.

[42] Lepourcelet M, Shivdasani RA (2002). Characterization of a novel mammalian Groucho isoform and its role in transcriptional regulation.. J Biol Chem.

[43] Marcal N, Patel H, Dong Z, Belanger-Jasmin S, Hoffman B (2005). Antagonistic effects of Grg6 and Groucho/TLE on the transcription repression activity of brain factor 1/FoxG1 and cortical neuron differentiation.. Mol Cell Biol.

[44] Blyth K, Cameron ER, Neil JC (2005). The RUNX genes: gain or loss of function in cancer.. Nat Rev Cancer.

[45] Cameron ER, Neil JC (2004). The Runx genes: lineage-specific oncogenes and tumor suppressors.. Oncogene.

[46] McLarren KW, Theriault FM, Stifani S (2001). Association with the nuclear matrix and interaction with Groucho and RUNX proteins regulate the transcription repression activity of the basic helix loop helix factor Hes1.. J Biol Chem.

[47] Javed A, Guo B, Hiebert S, Choi JY, Green J (2000). Groucho/TLE/R-esp proteins associate with the nuclear matrix and repress RUNX (CBF(alpha)/AML/PEBP2(alpha)) dependent activation of tissue-specific gene transcription.. J Cell Sci.

[48] Yarmus M, Woolf E, Bernstein Y, Fainaru O, Negreanu V (2006). Groucho/transducin-like Enhancer-of-split (TLE)-dependent and -independent transcriptional regulation by Runx3.. Proc Natl Acad Sci U S A.

[49] Torquati A, O'Rear L, Longobardi L, Spagnoli A, Richards WO (2004). RUNX3 inhibits cell proliferation and induces apoptosis by reinstating transforming growth factor beta responsiveness in esophageal adenocarcinoma cells.. Surgery.

[50] Chi XZ, Yang JO, Lee KY, Ito K, Sakakura C (2005). RUNX3 suppresses gastric epithelial cell growth by inducing p21(WAF1/Cip1) expression in cooperation with transforming growth factor {beta}-activated SMAD.. Mol Cell Biol.

[51] Yano T, Ito K, Fukamachi H, Chi XZ, Wee HJ (2006). The RUNX3 tumor suppressor upregulates Bim in gastric epithelial cells undergoing transforming growth factor beta-induced apoptosis.. Mol Cell Biol.

[52] Ito K, Liu Q, Salto-Tellez M, Yano T, Tada K (2005). RUNX3, a novel tumor suppressor, is frequently inactivated in gastric cancer by protein mislocalization.. Cancer Res.

[53] Goel A, Arnold CN, Tassone P, Chang DK, Niedzwiecki D (2004). Epigenetic inactivation of RUNX3 in microsatellite unstable sporadic colon cancers.. Int J Cancer.

[54] Sakakura C, Hasegawa K, Miyagawa K, Nakashima S, Yoshikawa T (2005). Possible involvement of RUNX3 silencing in the peritoneal metastases of gastric cancers.. Clin Cancer Res.

[55] Nakase Y, Sakakura C, Miyagawa K, Kin S, Fukuda K (2005). Frequent loss of RUNX3 gene expression in remnant stomach cancer and adjacent mucosa with special reference to topography.. Br J Cancer.

[56] Levanon D, Brenner O, Otto F, Groner Y (2003). Runx3 knockouts and stomach cancer.. EMBO Rep.

[57] Guo WH, Weng LQ, Ito K, Chen LF, Nakanishi H (2002). Inhibition of growth of mouse gastric cancer cells by Runx3, a novel tumor suppressor.. Oncogene.

[58] Brenner O, Levanon D, Negreanu V, Golubkov O, Fainaru O (2004). Loss of Runx3 function in leukocytes is associated with spontaneously developed colitis and gastric mucosal hyperplasia.. Proc Natl Acad Sci U S A.

[59] Harrington JM, Kolodner RD (2007). Saccharomyces cerevisiae Msh2–Msh3 Acts in Repair of Base∶Base Mispairs.. Mol Cell Biol.

[60] Baker SM, Harris AC, Tsao JL, Flath TJ, Bronner CE (1998). Enhanced intestinal adenomatous polyp formation in Pms2−/−;Min mice.. Cancer Res.

[61] Edelmann W, Yang K, Kuraguchi M, Heyer J, Lia M (1999). Tumorigenesis in Mlh1 and Mlh1/Apc1638N mutant mice.. Cancer Res.

[62] Harfe BD, Minesinger BK, Jinks-Robertson S (2000). Discrete in vivo roles for the MutL homologs Mlh2p and Mlh3p in the removal of frameshift intermediates in budding yeast.. Curr Biol.

[63] Allen T, van Tuyl M, Iyengar P, Jothy S, Post M (2006). Grg1 acts as a lung-specific oncogene in a transgenic mouse model.. Cancer Res.

[64] Dang J, Inukai T, Kurosawa H, Goi K, Inaba T (2001). The E2A-HLF oncoprotein activates Groucho-related genes and suppresses Runx1.. Mol Cell Biol.

[65] Levanon D, Goldstein RE, Bernstein Y, Tang H, Goldenberg D (1998). Transcriptional repression by AML1 and LEF-1 is mediated by the TLE/Groucho corepressors.. Proc Natl Acad Sci U S A.

[66] Brantjes H, Roose J, van De Wetering M, Clevers H (2001). All Tcf HMG box transcription factors interact with Groucho-related co-repressors.. Nucleic Acids Res.

[67] Daniels DL, Weis WI (2005). Beta-catenin directly displaces Groucho/TLE repressors from Tcf/Lef in Wnt-mediated transcription activation.. Nat Struct Mol Biol.

[68] Brett D, Pospisil H, Valcarcel J, Reich J, Bork P (2002). Alternative splicing and genome complexity.. Nat Genet.

[69] Leon C, Lobe CG (1997). Grg3, a murine Groucho-related gene, is expressed in the developing nervous system and in mesenchyme-induced epithelial structures.. Dev Dyn.

[70] Milili M, Gauthier L, Veran J, Mattei MG, Schiff C (2002). A new Groucho TLE4 protein may regulate the repressive activity of Pax5 in human B lymphocytes.. Immunology.

[71] Yang K, Edelmann W, Fan K, Lau K, Leung D (1998). Dietary modulation of carcinoma development in a mouse model for human familial adenomatous polyposis.. Cancer Res.

[72] Yang WC, Mathew J, Velcich A, Edelmann W, Kucherlapati R (2001). Targeted inactivation of the p21(WAF1/cip1) gene enhances Apc-initiated tumor formation and the tumor-promoting activity of a Western-style high-risk diet by altering cell maturation in the intestinal mucosal.. Cancer Res.

[73] Velcich A, Yang W, Heyer J, Fragale A, Nicholas C (2002). Colorectal cancer in mice genetically deficient in the mucin Muc2.. Science.

[74] Kucherlapati M, Yang K, Kuraguchi M, Zhao J, Lia M (2002). Haploinsufficiency of Flap endonuclease (Fen1) leads to rapid tumor progression.. Proc Natl Acad Sci U S A.

[75] Lin DP, Wang Y, Scherer SJ, Clark AB, Yang K (2004). An Msh2 point mutation uncouples DNA mismatch repair and apoptosis.. Cancer Res.

[76] Yang G, Scherer SJ, Shell SS, Yang K, Kim M (2004). Dominant effects of an Msh6 missense mutation on DNA repair and cancer susceptibility.. Cancer Cell.

[77] Wang Y, Putnam CD, Kane MF, Zhang W, Edelmann L (2005). Mutation in Rpa1 results in defective DNA double-strand break repair, chromosomal instability and cancer in mice.. Nat Genet.

