# Supplementary figures and images for: Novel Roles for MLH3 Deficiency and TLE6-Like Amplification in DNA Mismatch Repair-Deficient Gastrointestinal Tumorigenesis and Progression
Source: PLoS Genet. 2008 Jun 13;4(6):e1000092. doi: 10.1371/journal.pgen.1000092 (PMC2410297; doi:10.1371/journal.pgen.1000092)

## Slide 1
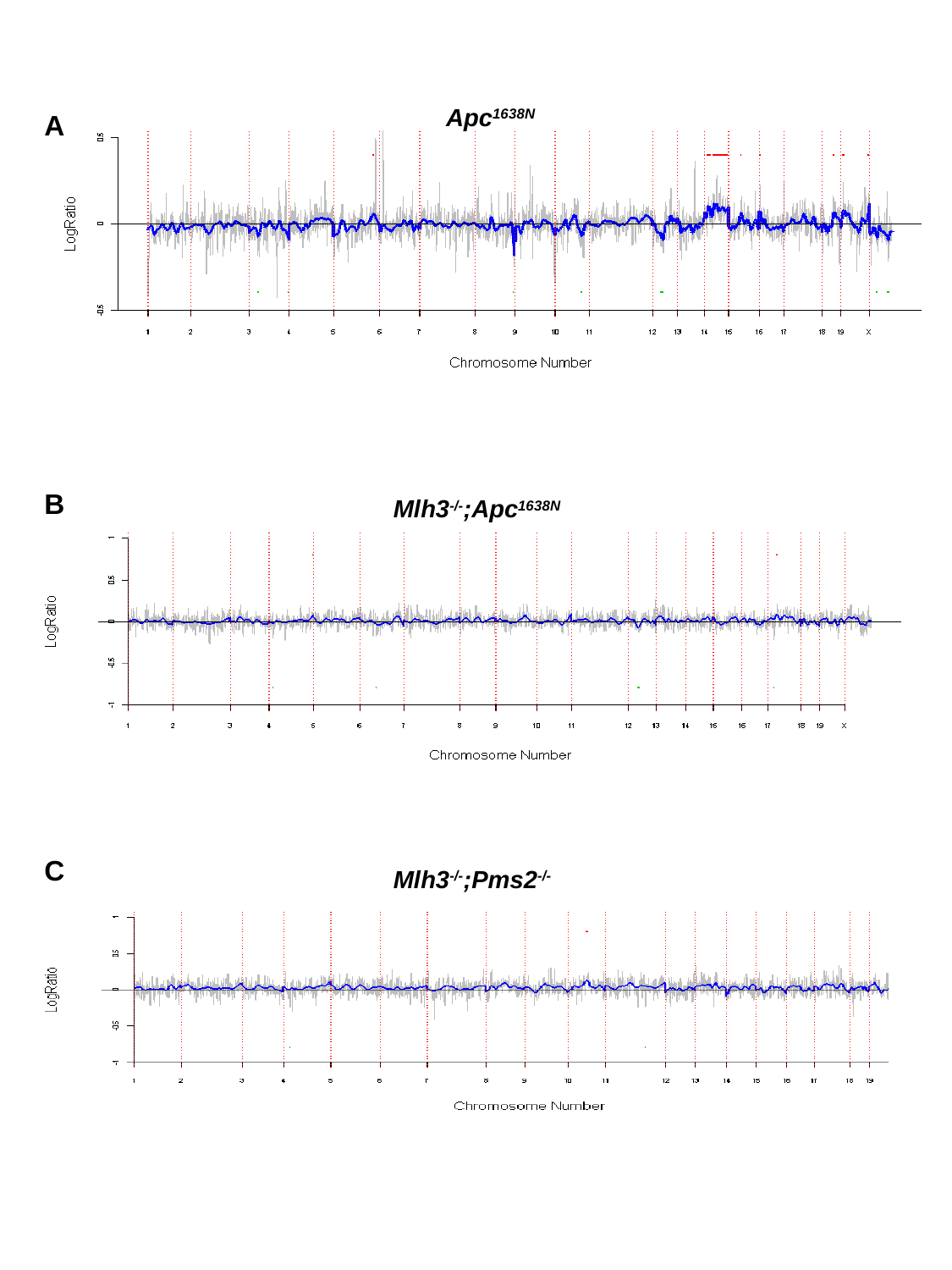

Apc1638N
A
B
Mlh3-/-;Apc1638N
C
Mlh3-/-;Pms2-/-

Supplement: Figures S1 — Array Comparative Genome Hybridization (aCGH) analysis of GI tumors. (A) Display of aCGH signal genome wide from a representative Apc1638N tumor. (B) Display of aCGH signal genome wide from a representative Mlh3−/−;Apc1638N tumor. (C) Display of aCGH signal genome wide from a representative Mlh3−/−;Pms2−/− tumor. (0.07 MB PPT) [file pgen.1000092.s001.ppt]

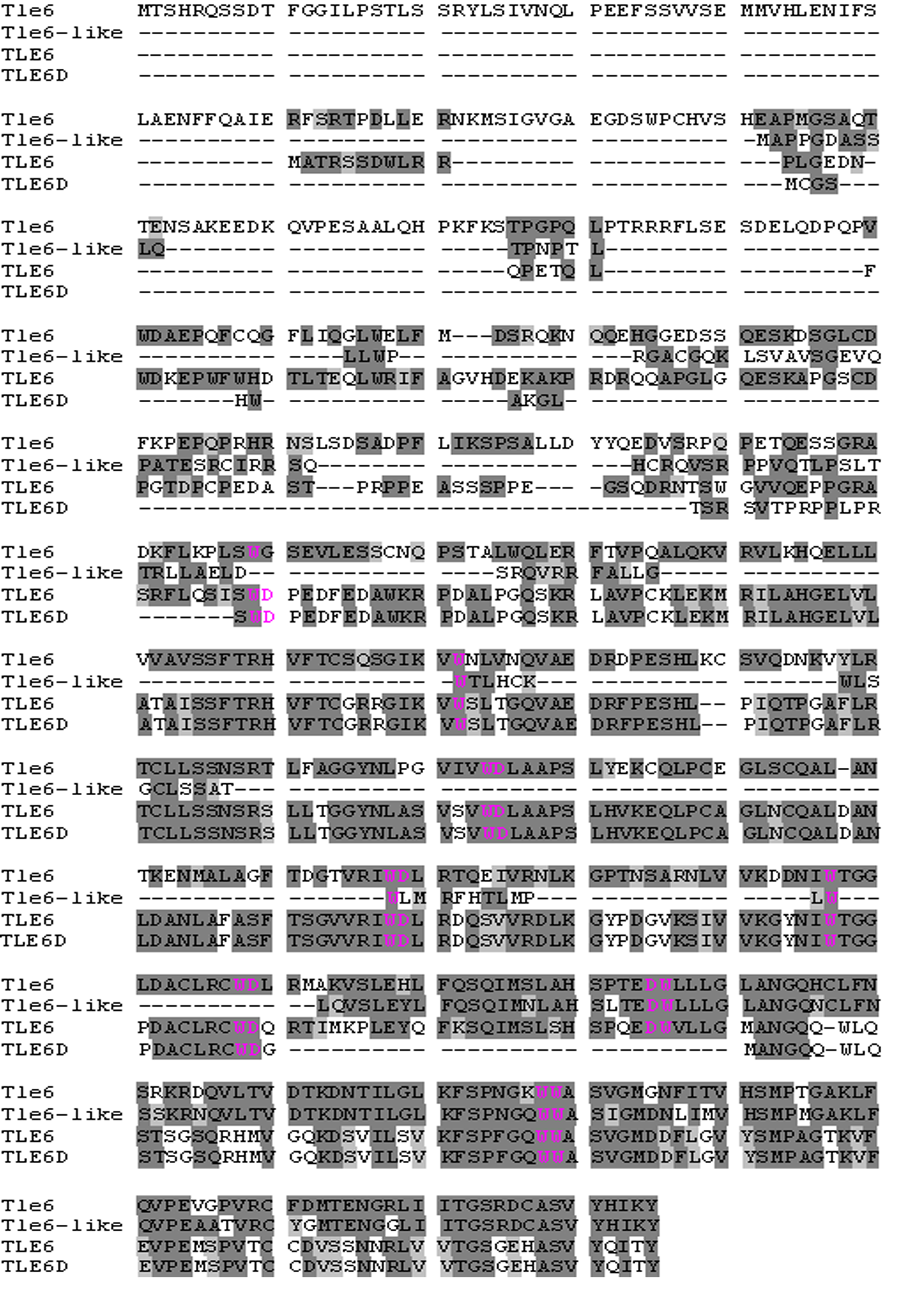

Supplement: Figure S2 — Protein sequences of TLE family. (1.95 MB PNG) [file pgen.1000092.s002.png]

## Slide 1
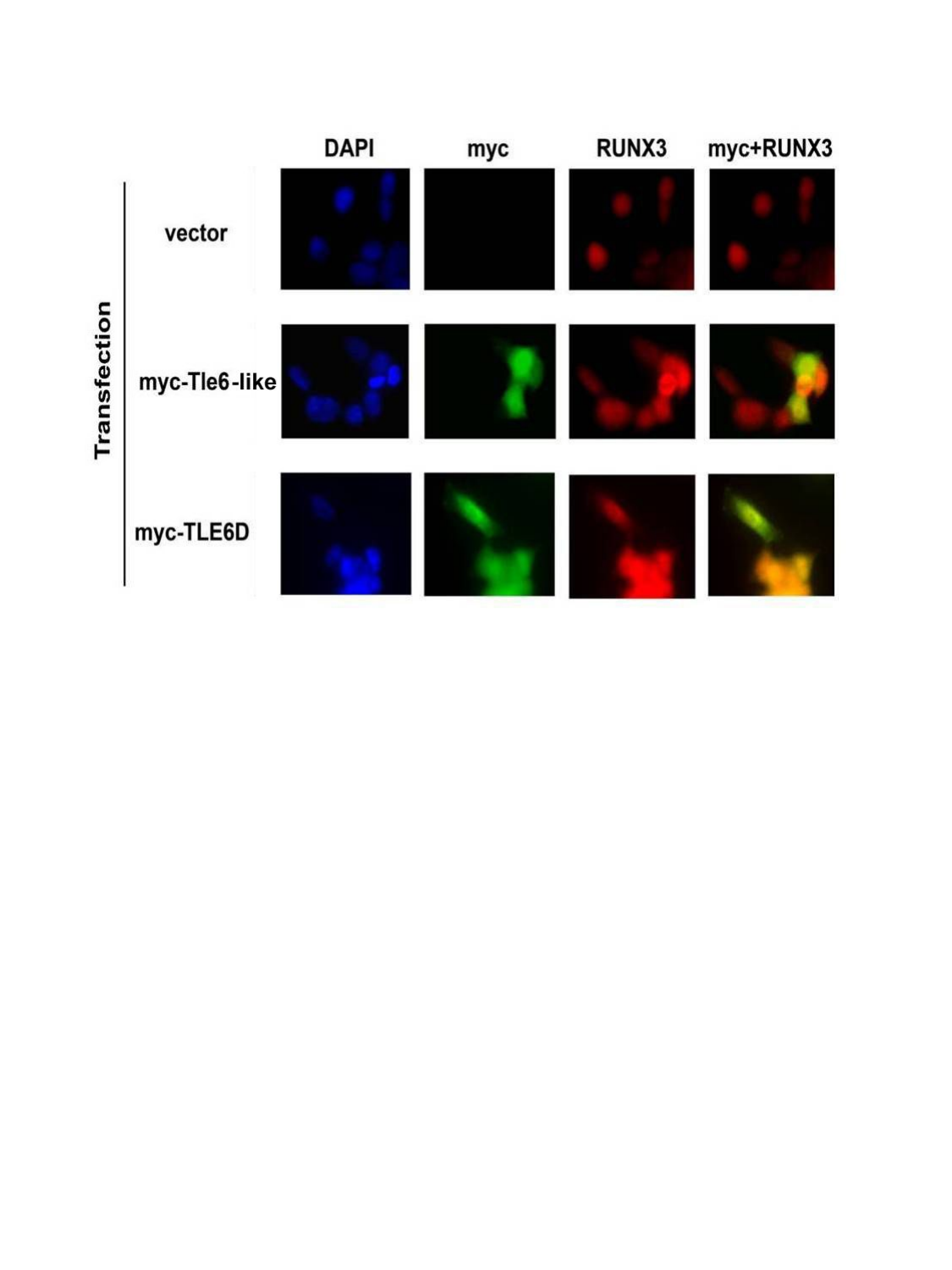

Supplement: Figure S3 — Cellular localization of endogenous RUNX3 and transfected Myc-epitope tagged Tle6-like in 293cells. Mouse monoclonal anti-myc and rabbit anti-RUNX3 were used. Secondary FITC-conjugated anti-mouse and Cy5-conjuaged anti-rabbit antibodies were used respectively. DAPI (4′,6-diamidino-2-phenylindole) staining indicates the nuclear location. (0.19 MB PPT) [file pgen.1000092.s003.ppt]
